# Retraction Note: Sepsis-induced elevation in plasma serotonin facilitates endothelial hyperpermeability

**DOI:** 10.1038/s41598-019-50843-4

**Published:** 2019-09-27

**Authors:** Yicong Li, Coedy Hadden, Anthonya Cooper, Asli Ahmed, Hong Wu, Vladimir V. Lupashin, Philip R. Mayeux, Fusun Kilic

**Affiliations:** 1 Departments of Biochemistry and Molecular Biology, University of Arkansas for Medical Sciences, Little Rock, Arkansas US; 2 Departments of Physiology University of Arkansas for Medical Sciences, Little Rock, Arkansas US; 3 Pharmacology and Toxicology, University of Arkansas for Medical Sciences, Little Rock, Arkansas US

Retraction of: *Scientific Reports* 10.1038/srep22747, published online 09 March 2016

This Article is being retracted.

The Editorial Office was alerted by the Authors to the errors in the assembly of Figure 5B and Figure 6A, for which the images showing actin control were swapped. Upon request of the Editorial Office, the Authors were not able to provide matching images of the original membranes for Figures 2, 5, and 6. Since the Authors are unable to provide the original data used to generate these published figures, the Editors are unable to guarantee the accuracy of the results.

Anthonya Cooper, Hong Wu, Vladimir V. Lupashin, and Philip R. Mayeux agree to the retraction. Yicong Li, Coedy Hadden, and Asli Ahmed could not be reached. Fusun Kilic disagrees with the retraction.

